# “Why won’t the ammonia go down?”: ammonia management while on continuous kidney replacement therapy

**DOI:** 10.1007/s00467-025-07006-7

**Published:** 2025-10-16

**Authors:** Michelle C. Starr, Jason Burnham, Michelle Voivoidas, Amy Wilson

**Affiliations:** 1 Division of Nephrology, Department of Pediatrics, Indiana University School of Medicine, Suite 2000A, Indianapolis, IN 410 W 1046202 USA; 2 Division of Critical Care Medicine, Department of Pediatrics, Indiana University School of Medicine, Indianapolis, IN USA

**Keywords:** Ammonia, Hyperammonemia, Kidney replacement therapy, Glucose, Dialysis

## Abstract

**Background:**

Kidney replacement therapy (KRT) is commonly used to treat critically ill children for a variety of reasons, including hyperammonemia. KRT management in children with hyperammonemia not due to inborn errors of metabolism is challenging.

**Case presentation:**

We report a complex case of hyperammonemia in a 17-year-old critically ill female patient, emphasizing the challenges of management in a pediatric intensive care setting. Despite the initiation of kidney replacement therapy (KRT) and progressive increases in the prescribed dialytic dose, the patient’s ammonia levels continued to escalate. This prompted a reevaluation of her metabolic needs, with a focus on optimizing glucose delivery to facilitate ammonia metabolism and dialytic clearance. Adjustments to increase the delivered glucose, along with careful monitoring of glucose removal during KRT, ultimately led to the stabilization of her ammonia levels.

**Conclusion:**

This case underscores the intricate interplay between metabolic support and dialytic strategies in the management of hyperammonemia. The use of a glucose delivery calculator is proposed. This case highlights the need for individualized, dynamic approaches in critically ill pediatric patients.

**Supplementary Information:**

The online version contains supplementary material available at 10.1007/s00467-025-07006-7.

## Initial case presentation

A 17-year-old 40-kg young woman with a history of severe aplastic anemia status post-bone marrow transplant (BMT) 1 month prior was transferred to the pediatric intensive care unit (PICU) with methicillin-resistant *Staphylococcus aureus* bacteremia from the BMT inpatient unit. Her post-BMT course had been complicated by moderate vaso-occlusive disease treated with defibrotide and evidence of thrombotic microangiopathy treated with eculizumab. She also had prolonged bone marrow suppression requiring almost daily blood product transfusions.

**Fig. 1:**
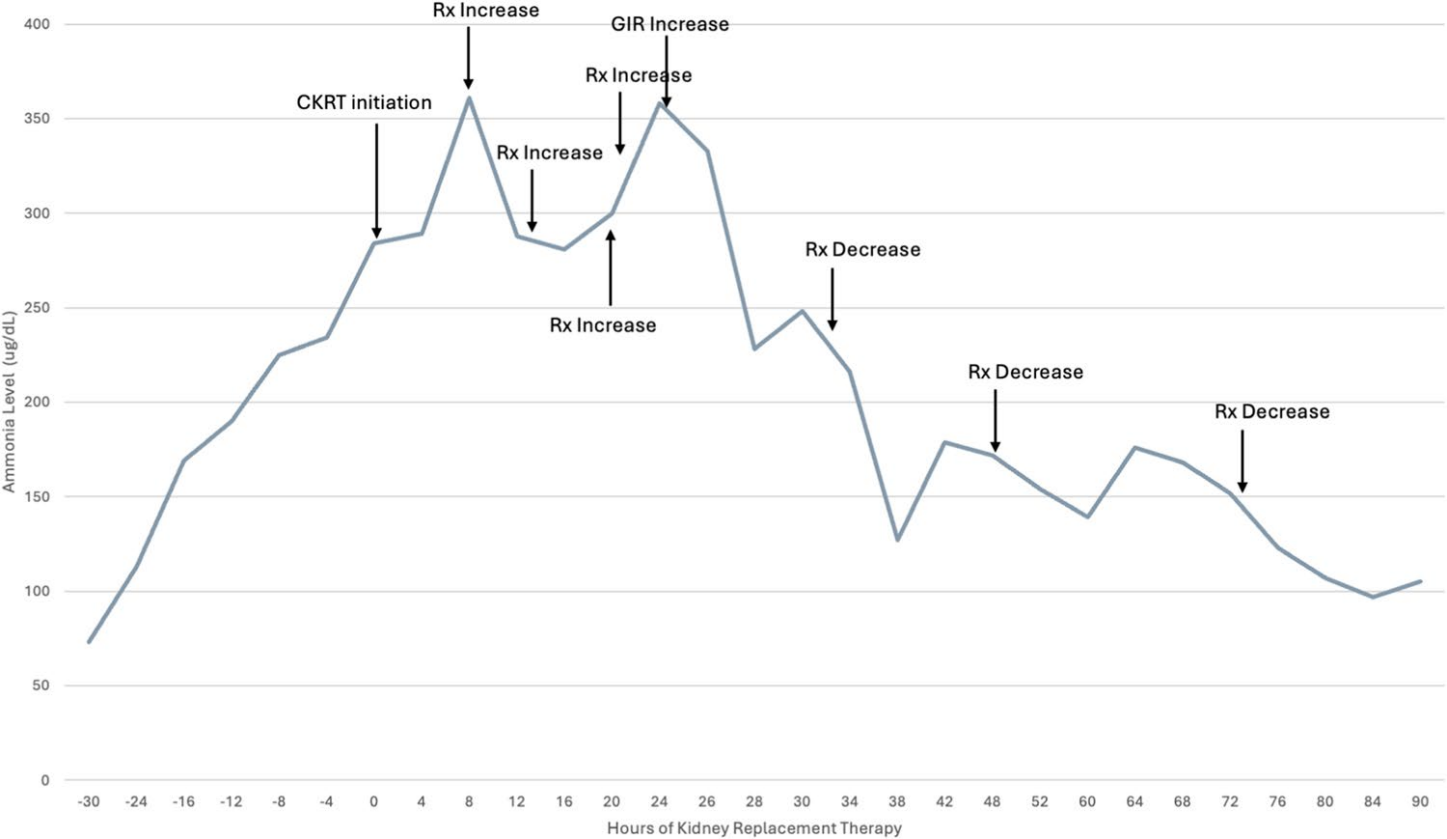
Ammonia levels during the course of intensive care unit admission for the patient, with denotations of CKRT initiation, prescription increases/decreases, and increase in glucose infusion rate (GIR)

Her clinical decompensation progressed to include stage 3 oliguric acute kidney injury (peak creatinine of 1.99 mg/dL) with 17% fluid overload. She received a 1.5 mg/kg dose of furosemide and did not increase her urine output by more than 1 mL/kg/h (furosemide non-responder). She had worsening liver injury, concerning for hepatocellular necrosis (AST/ALT > 2000 units/L and INR 1.69) and hypoxemic respiratory failure requiring intubation and mechanical ventilation. She developed altered mental status in the setting of this decompensation and was noted to have an ammonia of 194 µg/dL (Fig. 1). While the differential diagnosis for her altered mental status was broad, it included sepsis-associated encephalopathy, medication side effect, hyperammonemia-related encephalopathy, and uremia. The PICU consulted pediatric nephrology for consideration of kidney replacement therapy (KRT) given her altered mental status, oliguric AKI, and elevated ammonia level.

## Discussion of initial management and decision to start KRT

Kidney replacement therapy (KRT) is commonly used to treat critically ill children for a variety of indications, including but not limited to fluid overload, electrolyte derangements, uremia, toxin removal, and hyperammonemia [1]. Hyperammonemia, defined as elevated plasma ammonia levels in the blood, is a rare but urgent indication for the provision of KRT [2]. Hyperammonemia is commonly defined as > 100 µmol/l in neonates and > 50 µmol/l in older children [3]. It is essential that when there is a clinical concern for hyperammonemia, blood is drawn from a free-flowing sample, placed on ice, and immediately processed and analyzed, as alterations from this standard approach may impact the accuracy of the values [4, 5]. Ammonia is produced by the catabolism of amino acids through glutamine dehydrogenase activity in the brain, liver, kidney, and pancreas. In normal states, most ammonia enters the urea cycle, is converted into glutamine, or is excreted as urea in the urine. Hyperammonemia occurs when there is a mismatch between ammonia production and capacity to excrete waste due to either liver or kidney dysfunction [6].

Clinical presentation of hyperammonemia depends on the age of the patient but can include vomiting, loss of appetite, and lethargy. As ammonia levels rise, neurologic symptoms can progress to include seizures and, if treatment is not expeditiously provided, coma and death. The pathophysiology of altered mental status in children with hyperammonemia is due to increased levels of ammonia in the brain, leading to metabolism to glutamine by astrocytes, resulting in increased intracellular osmolality, edema, and inflammatory cytokine production.

The common clinical presentations, evaluation, and management of hyperammonemia in the newborn or infant with suspected or confirmed inborn errors of metabolism (IEM) have been discussed in previous literature [3, 6–8]. The overwhelming majority of existing consensus recommendations for management of hyperammonemia are based on either small single-center series or the experience of infants with IEM [9]. No consensus guidelines exist regarding the non-dialytic management, timing of continuous KRT (CKRT), nor CKRT clearance prescription approaches in children with non-IEM-related hyperammonemia [3, 6–8].

The remainder of this discussion will be focused on hyperammonemia in critically ill patients with other—often multifactorial—causes of non-IEM hyperammonemia. While there is no clearly defined “threshold” of hyperammonemia at which KRT is indicated in such patients, it is often the case that ammonia management is but one of several considerations to be weighed in a decision to start KRT. In this patient, with altered mental status and an ammonia level of almost 200 µg/dL, complicated by superimposed AKI, fluid overload, and an ongoing need for high-volume blood product and medication administration, the utility of more conservative management strategies is limited, and initiation of KRT is certainly clinically indicated, with reduction of ammonia among the goals of treatment [10, 11].

## Initial CKRT course

Given her overall worsening clinical status, the decision was made to start the patient on CKRT using hemo-diafiltration (CVVHDF). Using a PrisMax (Baxter Internation, Deerfield, Illinois) with a HF-1000 filter (Baxter Internation, Deerfield, Illinois), her initial prescription was for a prescribed total clearance of 1400 ml/h (roughly 35 ml/kg or 2000 ml/1.73 m^2^) divided into 600 ml/h dialysis fluids, 400 ml/h pre-filter replacement fluids, and 400 ml/h post-filter replacement fluids. Due to her liver dysfunction, citrate anticoagulation was provided in accordance with a local “high-risk citrate accumulation” protocol, which uses a lower rate of citrate delivery and targets a higher circuit ionized calcium (0.4–0.6 mmol/L) protocol to limit citrate delivery and citrate accumulation. Initial dialysis fluids were Phoxilium (Gambro Lundia AB, Lund, Sweden) (4 meq/L of potassium and 1 mmol/L phosphorus).

Given her hyperammonemia, liver dysfunction, and fluid overload, nutritional and fluid management initially focused on limiting protein and fluid intake. On the day of CKRT initiation, her parenteral nutrition was held, and fluids administered included only D10 and other necessary medications.

## Discussion of CKRT prescription and initial management

This patient was initially managed with a “standard” KRT approach using CVVHDF. In the context of her hyperammonemia, one element of her management merits further discussion and consideration—that of her glucose delivery and other supportive measures to promote alternative pathways of ammonia removal and suppress ongoing ammoniagenesis.

The patient’s glucose infusion rate (GIR) on the day of CKRT initiation was 1.02 mg/kg/min. GIR is a critical parameter that quantifies the amount of glucose delivered to a patient per unit time and body weight and is calculated using the formula:

$$GIR=\frac{Glucose\;concentration\left(\frac g{dL}\right)x\;Infusion\;rate\left(\frac{mL}{hr}\right)x100}{Body\;weight\left(kg\right)x60}$$

Existing consensus recommendations for the management of hyperammonemia uniformly mention targeting a high GIR [7, 9]. In infants with IEM, high rates of glucose fluids are commonly administered. However, in older children, this is more difficult and often is not universal practice. There are often concerns about the complications of increasing GIR and the resulting potential hyperglycemia. However, hyperglycemia can be managed with insulin or other medical strategies when it occurs.

Increased glucose delivery, as can be quantified by the GIR, directly reduces hyperammonemia by enhancing the body’s metabolic capacity to detoxify ammonia. Elevated glucose levels stimulate insulin secretion, which reduces proteolysis and decreases ammonia generation through catabolism of amino acids. Simultaneously, glucose upregulates urea cycle activity in the liver, promoting conversion of ammonia into urea for excretion. Glucose additionally increases tricarboxylic acid (TCA) cycle activity, upregulating the production of α-ketoglutarate and other intermediaries that aid ammonia conversion to both glutamate and glutamine, effectively sequestering ammonia in the liver [6]. In this patient, at the time of identification of high ammonia levels, attention should have been directed to increasing the GIR to a goal of > 5 mg/kg/min (ideally closer to 7–8 mg/kg/min), with resulting hyperglycemia addressed with the addition of an insulin infusion if needed.

## Subsequent CKRT course and further investigations

**Fig. 2:**
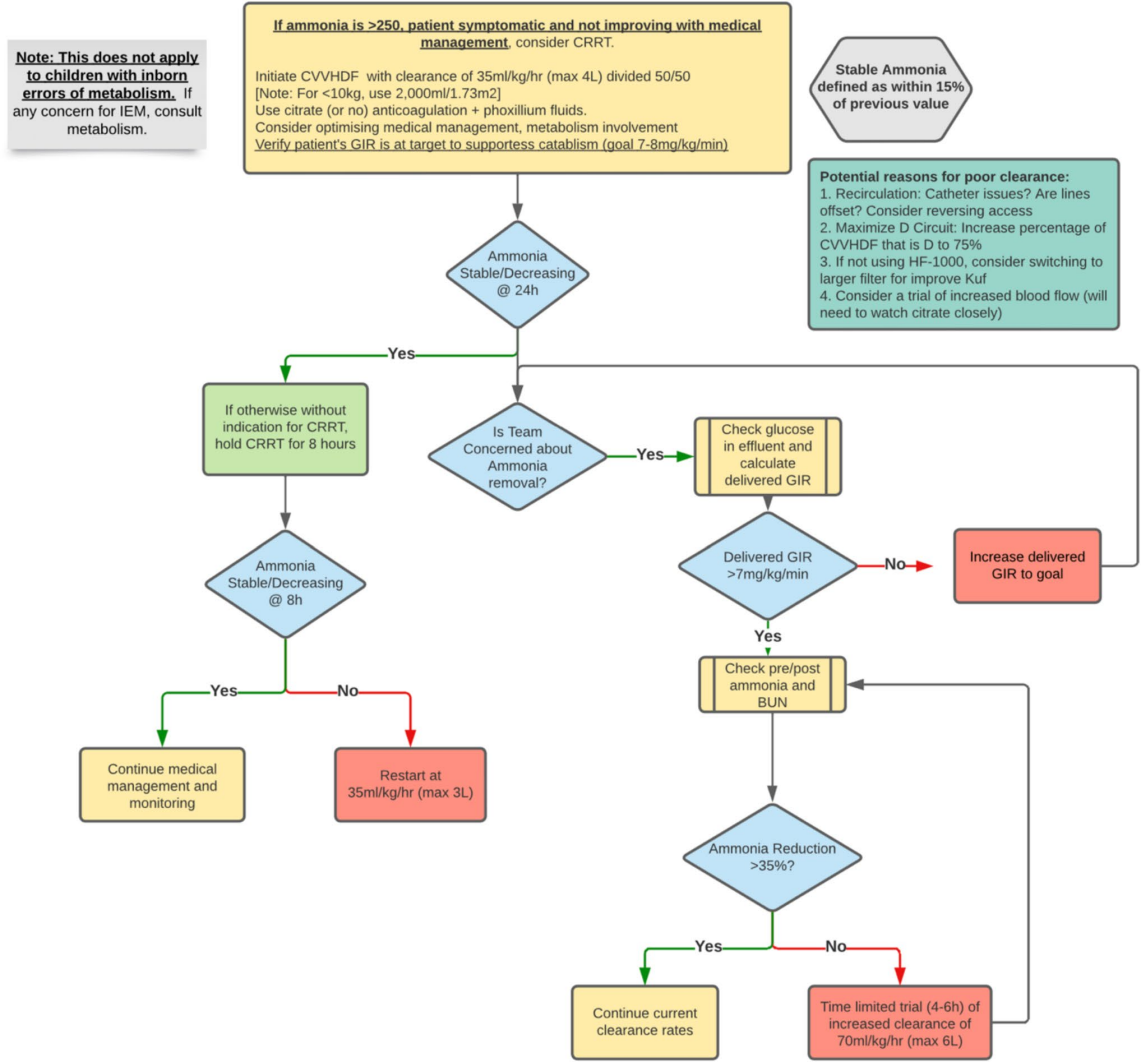
Local algorithm for adjustment and management of CKRT in children with secondary (non-inborn errors of metabolism) hyperammonemia

Despite the initiation of CKRT, the ammonia level continued to increase. Several stepwise increases in prescribed dialysis dose were performed in close succession; however, the ammonia level continued to rise despite a peak delivered dialysis dose of 3000 ml/h (75 ml/kg or 6000 ml/1.73 m^2^). Further investigations included the assessment of single-pass  ammonia removal (drawn along with BUN and comparing post-filter levels to a patient level in a similar manner to what would be done to assess re-circulation),  which demonstrated appropriate and adequate single-pass removal of ~40% (Fig. 2.)

**Fig. 3:**
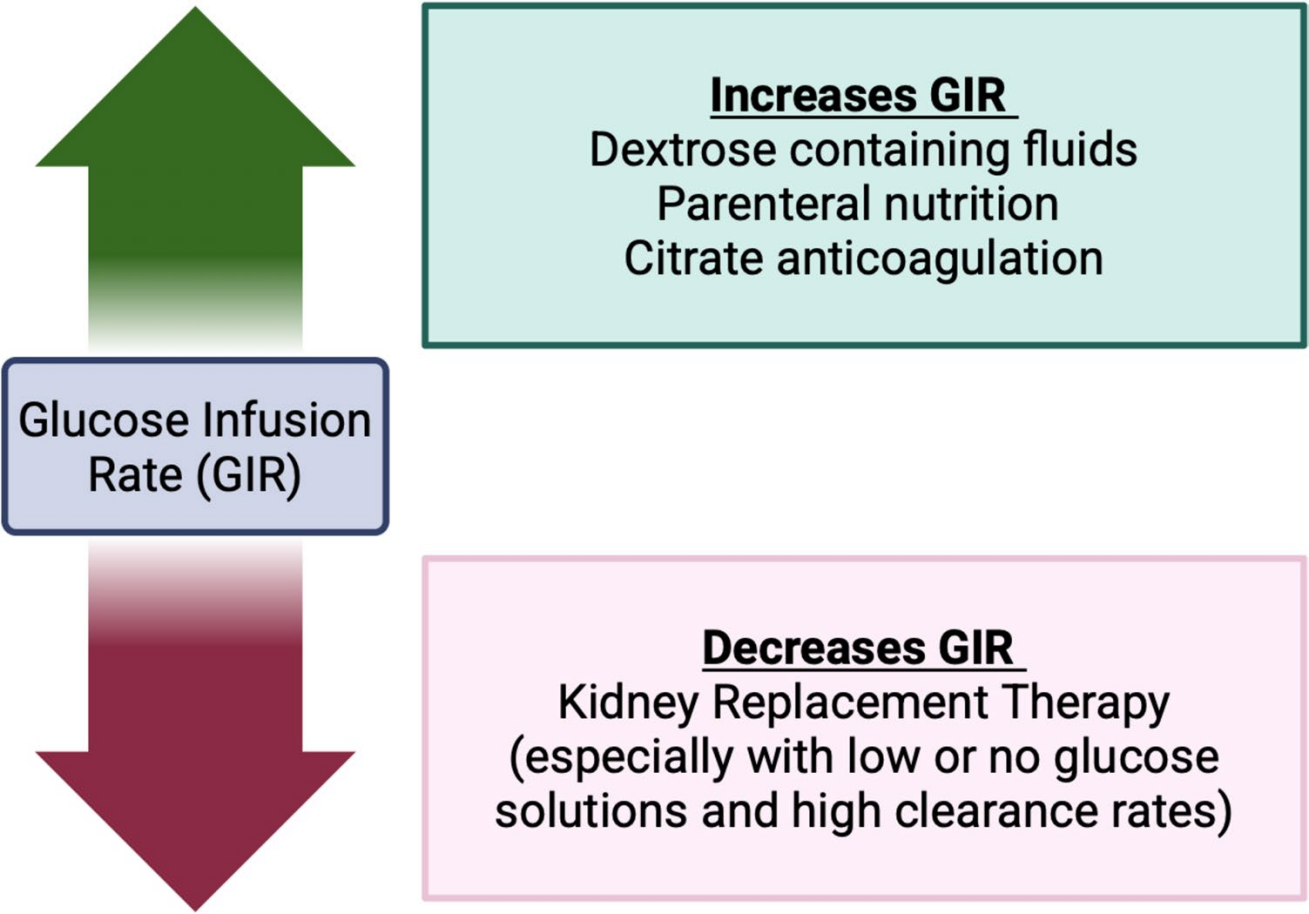
Factors influencing glucose infusion rate in patients on kidney replacement therapy

At approximately 24 h after CKRT initiation, in the face of worsening ammonia levels despite robust clearance and the absence of recirculation, the nephrology team undertook a comprehensive review of the patient’s medication, nutrition, and fluid intake. No medications contributing to ammoniagenesis were noted (Supplemental Table 1). It was noted that glucose delivery (with dextrose-containing fluids) remained low, and parenteral nutrition remained held, given the priority of limiting protein in the setting of acute hyperammonemia. Further investigations were performed, including measurement of effluent glucose (Fig. 3).

## Discussion of subsequent CKRT course and further investigations

When laboratory findings, such as an elevated ammonia level, do not respond to what should be adequate CKRT management, further investigations are necessary (Fig. 2). The first investigations performed were the following: (1) evaluation of single-pass ammonia removal to assess for inadequate ammonia removal and (2) assessment of dialysis access recirculation.

$$Single\;pass\;removal=\frac{Concentration\;at\;inlet-Concentratration\;at\;outlet}{Concentration\;at\;inlet}\times100$$

$$Dialysis\;access\;recirculation=\frac{Peripheral\;BUN-Pre\;CKRT\;BUN}{Peripheral\;BUN-Post-CKRT\;BUN}\times100$$

Our evaluation indicated that we had appropriate single-pass removal and minimal evidence of recirculation (7%), suggesting that the issue was the ongoing generation of ammonia as opposed to inadequate removal or recirculation [3]. The patient’s liver dysfunction had not worsened (stable AST/ALT ~ 2000 Units/L and INR 1.52), and her clinical course had slightly improved, such that neither of these factors appeared to be contributing to worsening ammonia levels despite high-dose CKRT.

We then turned our focus to better understand other factors promoting ongoing ammonia generation. Specifically, we evaluated whether the glucose level that our patient was exposed to was adequate to suppress or minimize ongoing ammonia generation. At the time of this reassessment, the patient’s total glucose delivery now included her dextrose-containing intravenous fluids and dextrose from citrate regional anticoagulation for CKRT. Citrate regional anticoagulation solution (acid citrate dextrose formula A or ACD-A) contains 2.45 g of dextrose per 100 ml citrate solution [12]. A study of critically ill adults treated with continuous veno-venous hemodiafiltration (blood flow of 100 ml/min, 2000 ml/h total prescribed clearance) described glucose uptake of 567 mmol/day with ACD-A anticoagulation contributing to substantial bioenergetic gain [13]. We therefore calculated her current GIR including both her parenteral dextrose delivery (D10 fluids) as well as her citrate infusion (Fig. 3).

$$GIR=GIR\;for\;dextrose\;containing\;fluids\;and\;PN+\frac{ADCA\;rate\;\times2.45\;\times1000}{Body\;weight\left(kg\right)\;\times60\times100}$$

On the opposite side, CKRT, especially at high dialytic doses as seen in the management of this patient, is known to result in profound loss of micronutrients and protein [14]. As many standardly used dialysis fluids (such as Phoxilium which is used primarily at our institution) do not contain dextrose, glucose removal while on CKRT can be significant and clinically important (Table 1). This loss may be more profound at higher doses and when using fluids with low concentrations of glucose. We calculated glucose removal from the effluent by measuring glucose concentration in effluent and calculating removal based on effluent dose. We estimated total effluent as the hourly replacement fluid rate plus the hourly dialysis fluid rate plus the average CKRT fluid removal rate (averaged over the prior 6 h).

**Table 1:** Glucose content of commonly used CKRT solutions

Solution name	Manufacturer	Glucose concentration
PRISMASOL BGK	Gambro, Baxter	
BGK 4/2.5 (calcium formula)		100 mg/dL
BGK 4/3.5 (calcium formula)		100 mg/dL
BGK 0/2.5 (calcium formula)		100 mg/dL
BGK 4/0/1.2 (calcium-free formula)		100 mg/dL
BGK 2/0 (calcium-free formula)		100 mg/dL
BGK 4/0 (calcium-free formula)		100 mg/dL
**BK 0/0/1.2 (calcium-free formula)**		**0 mg/dL**
PRISMASATE	Gambro, Baxter	
BGK 4/2.5 (calcium formula)		110 mg/dL
BK 0/3.5 (calcium formula)		**0 mg/dL**
BGK 2/0 (calcium-free formula)		110 mg/dL
BGK 4/0/1.2 (calcium-free formula)		110 mg/dL
B22GK 4/0 (calcium-free formula)		110 mg/dL
BK 2/0 (calcium-free formula)		**0 mg/dL**
PHOXILLUM	Gambro	
BK 4.25		**0 mg/dL**
B22K4/0		**0 mg/dL**
PureFlow Bicarbonate Solution	Fresenius Medical Care	
RFP-400, 401, 402, 403, 404, 406, 453, 454, 456		100 mg/dL

$$Glucose\;removal=\frac{Glucose\;effluent\;concentration\left(\frac{mg}{dL}\right)\times Total\;estimated\;effluent\left(\frac{mL}{hr}\right)}{Body\;weight\left(kg\right)x60}$$

Based on these calculations, the patient’s prescribed GIR was found to be 2.6 mg/kg/min, once accounting for both her D10-containing fluids and ADC-A infusion. However, given the high prescribed CKRT clearance and an effluent glucose concentration of 175 mg/dL, the total estimated glucose effluent rate was 2.2 mg/kg/min. Therefore, the net delivered GIR was only 0.4 mg/kg/min. Given this additional information regarding the “delivered GIR,” the patient’s GIR was subsequently increased by Y-ing in dextrose-containing fluids (D20) directly to the patient to increase the delivered glucose. Following this change, and with no additional increase in dialysis prescription, the ammonia began to decline (Fig. 1).

## Conclusion CKRT course and patient outcome

Over the next 24–48 h, the patient’s ammonia level continued to normalize. CKRT prescription was slowly decreased to standard prescribed dosing (35 ml/kg) over the next 24 h in a stepwise manner (Fig. 1). There was slow improvement in the patient’s fluid overload and acute kidney injury, which allowed for liberation from CKRT following 5 days of therapy with no rebound increase in ammonia levels. However, the patient’s multiple underlying disease processes continued to worsen. She died on ICU Day 10 from overwhelming infection and complications of her underlying illness.

## Conclusion

We present a challenging case of a 17-year-old young woman with hyperammonemia in the setting of critical illness. Despite KRT initiation and escalating prescribed dialytic dose, the patient’s ammonia levels continued to rise, prompting further investigations and adjustments in glucose delivery to enhance ammonia management. Increasing delivered glucose and quantitatively assessing glucose removal via KRT led to significant improvement in ammonia levels (Table 2). This case highlights the complexity and multi-modal nature of managing hyperammonemia in critically ill pediatric patients.

**Table 2:** Key management points^a^

In children with hyperammonemia, target glucose infusion rate of 7–8 ml/kg/min before starting CKRT whenever possible [2]
Reduce protein intake temporarily in the setting of severe hyperammonemia [3]
Appreciate that high clearance rates of CKRT can promote the removal of glucose [3]
If ammonia continues to rise after CKRT initiation, consider assessing single pass removal of ammonia [3]
If persistent hyperammonemia is not explained by insufficient dialysis (e.g. recirculation, etc.), calculate delivered glucose infusion rate by assessing effluent glucose and calculating glucose removal rate [4]

^a^Quality Rating Scheme for Studies and Other Evidence modified from Oxford Center for Evidence-based medicine per journal standards with ratings from 1 (properly powered and conducted randomized clinical trial; systematic review with meta-analysis) to 5 (opinion of respected authorities; case reports)

## Supplementary Information

Below is the link to the electronic supplementary material.

ESM1 (DOCX 14.5 KB)

## Data Availability

None
