# Supplementary material for: “Why won’t the ammonia go down?”: ammonia management while on continuous kidney replacement therapy
Source: Pediatr Nephrol. 2025 Oct 16;41(4):1213–9. doi: 10.1007/s00467-025-07006-7 (PMC12953408; doi:10.1007/s00467-025-07006-7)
Supplement: Supplementary file 1 — (DOCX 14.5 KB) [file 467_2025_7006_MOESM1_ESM.docx]

**Supplemental Table 1.** Drugs implicated in contributing to ammoniagenesis

| Medication Class | Examples |
| --- | --- |
| Anesthetics | Enflurane, halothane |
| Anticonvulsants | Carbamazepine, lamotrigine, phenobarbitone, phenytoin, primodone, topiramate, valproate, zonisamide |
| Chemotherapeutics | Asparaginase, fluorouracil, cyclophosphamide, cytarabine, etoposide, oxaliplatin, L- vincristine |
| Diuretics | Acetazolamide |
| Immune modulating therapy | Sunitinib, rituximab, regorafenib |
| Pain Management | Barbiturates, haloperidol, narcotics, salicylates |
| Steroids |  |
| Other | Glycine gel, ribavirin, sulfadiazine, tranexamic acid |

Note: Adapted from Belanger-Quintana A et al. [7]
